# A Rare Case of Pancreatic Neuroendocrine Tumor with Intraductal Extension in the Dorsal Duct of a Pancreas Divisum

**DOI:** 10.3390/reports9020104

**Published:** 2026-03-28

**Authors:** Salvatore Crucillà, Asia Berlato, Stefano Francesco Crinò, Luca Landoni, Maria Cristina Conti Bellocchi

**Affiliations:** 1 Gastroenterology and Digestive Endoscopy, Department of Medicine, University of Verona, 37134 Verona, Italy; salvatore.crucilla@univr.it (S.C.); stefanofrancesco.crino@aovr.veneto.it (S.F.C.); 2 Department of Surgery, Pancreas Institute, University and Hospital Trust of Verona, 37134 Verona, Italy; luca.landoni@aovr.veneto.it

**Keywords:** pancreatic neuroendocrine tumor, intraductal growth, pancreas divisum, endoscopic ultrasound, DAXX/ATRX

## Abstract

**Background and Clinical Significance:** Pancreatic neuroendocrine tumors (pNETs) rarely exhibit intraductal growth, a pattern that may mimic intraductal papillary mucinous neoplasms (IPMNs) or pancreatic ductal adenocarcinoma (PDAC). Preoperative recognition is challenging, particularly when associated with anatomic variants such as pancreas divisum. **Case Presentation:** A 63-year-old man with a history of pancreatic duct dilation presented with pruritus, weight loss, and lymphadenopathy. Cross-sectional imaging revealed a cephalopancreatic mass with upstream ductal dilatation. EUS demonstrated a hypervascular lesion with intraductal extension into the dorsal duct in the setting of pancreas divisum. EUS-FNB confirmed a well-differentiated pNET (G1) with loss of DAXX expression and preserved ATRX. Ga-68 PET/CT showed intense uptake in the primary lesion and lower-grade uptake in two additional nodules, later proven non-neoplastic. A multidisciplinary tumor board recommended preoperative optimization with somatostatin analog therapy and supervised weight reduction, followed by pylorus-preserving duodenocephalopancreatectomy. Final pathology confirmed NET G1 with intraductal growth and full concordance with preoperative EUS-FNB findings. **Conclusions:** in this case, a pNET showed intraductal growth within the dorsal duct in the context of pancreas divisus, further expanding the range of its reported presentations. It underscores the diagnostic value of EUS-FNB for morphologic, proliferative, and molecular characterization, and highlights the importance of multidisciplinary evaluation in guiding preoperative optimization and tailored surgical management.

## 1. Introduction and Clinical Significance

Pancreatic neuroendocrine tumors (pNETs) are the second-most common epithelial neoplasms of the pancreas, accounting for approximately 1–2% of all pancreatic tumors [1]. They are classified as well-differentiated neuroendocrine tumors (NETs) or poorly differentiated neuroendocrine carcinomas (NECs), based on morphology and proliferative index [2,3]. NETs are usually indolent, whereas NECs are aggressive with high Ki-67 index.

Approximately 80% of pNETs are non-functioning, presenting with nonspecific symptoms such as abdominal pain, pruritus, or jaundice. [4] Intraductal growth into the main or accessory pancreatic duct is exceptionally uncommon and may mimic intraductal papillary mucinous neoplasms (IPMNs) or pancreatic ductal adenocarcinomas (PDAC). Reported cases of pNETs with growth along the duct are summarized in Table 1 [1,5,6,7,8,9,10]. We report a unique case of pNET with intraductal growth in the dorsal duct within a pancreas divisum, a presentation not previously documented.

## 2. Case Presentation

### 2.1. Patient Information

A 63-year-old overweight man (BMI 34.6 kg/m^2^) presented to his general practitioner (GP) with diffuse pruritus and multiple lymphadenopathies that had been present for approximately one month. Jaundice was not present, and routine laboratory tests were within normal limits. Other symptoms, such as flushing, diarrhea, or episodes of hypoglycemia, were also absent. He had lost approximately 13 kg over nine months while following a restrictive diet for weight control due to obesity. Bowel habits and diuresis were normal, and appetite was preserved. The patient was a former smoker (40 pack-years, quit 25 years prior) and abstained from alcohol. His medical history included hypertension, well-controlled type 2 diabetes mellitus treated with oral antidiabetic agents, and hypercholesterolemia. Two years earlier, an MRI had been performed as part of the diagnostic workup for nonspecific abdominal pain, incidentally demonstrating dilation of the main pancreatic duct (5 mm) and prompting a recommendation for a 12-month follow-up MRI, which was not carried out.

### 2.2. Clinical Findings

Pancreatic enzymes, liver function tests, and cholestatic indices were within normal limits. A contrast-enhanced CT confirmed persistent ductal dilation and mild enlargement of the pancreatic head. A subsequent MRI revealed a solid, hypoenhancing mass (38 × 27 mm) located between the head and uncinate process, with restricted diffusion and upstream ductal dilatation (up to 9 mm). No biliary dilatation or lymphadenopathy was observed. The radiologic features raised suspicion for a degenerated IPMN, and the patient was therefore referred to our center for further characterization with Endoscopic UltraSound (EUS).

### 2.3. Diagnostic Assessment

**Figure 1:**
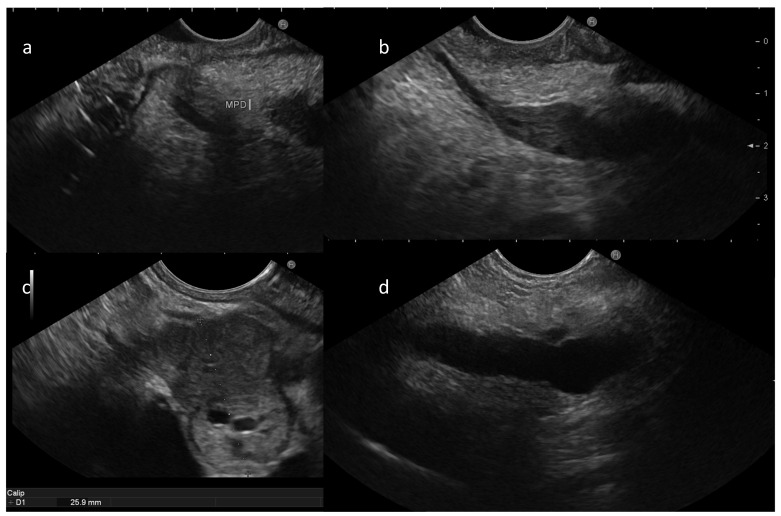
EUS examination documented pancreas divisum with no communication between ventral and dorsal part of the main pancreatic duct (MPD) (**a**) and presence of tissue alongside the dorsal duct (**b**) communicating with a 25 mm bilobated solid lesion (**c**), determining upstream main pancreatic duct dilatation (**d**).

EUS revealed an inhomogeneous, hypervascular lesion with intraductal growth within the dorsal duct, consistent with pancreas divisum (Figure 1). The lesion was located a few centimeters from the minor papilla and appeared as a bilobed hypervascular nodule measuring 25 mm (Figure 1c). Endoscopic UltraSound-guided fine-needle biopsy (EUS-FNB) was performed using a 22G Franseen needle (Acquire, Boston ScientificCorp, Boston, MA, USA). Histopathologic evaluation described fragments of epithelial neuroendocrine neoplasm (NEN). On immunohistochemical analysis: expression of DAXX was lost while ATRX was preserved, and Ki-67 index was 2-3%, which confirmed a well-differentiated NET G1. 

**Figure 2:**
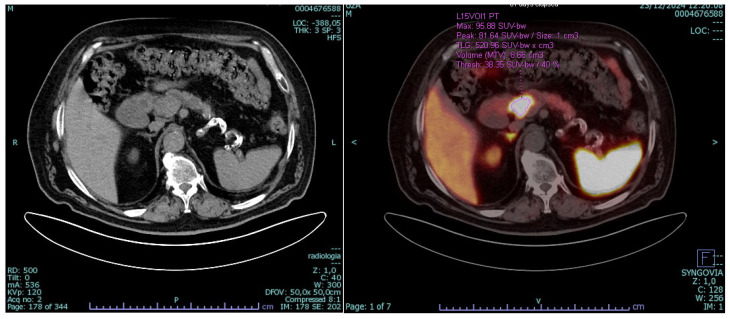
Gallium-68 PET/CT showing intense uptake (SUV max 95) in the cephalopancreatic lesion.

To complete the staging, Gallium-68 PET/CT showed a cephalopancreatic lesion with intense radiotracer uptake (SUV max 95, Figure 2). Moreover, a lower-grade uptake in two additional small pancreatic nodules (SUV 25 and 10) was documented, located in the body and the tail, later proven not to be NETs. No extra-pancreatic disease was detected. It is worth noting that EUS had been performed prior to the Ga-68 PET/CT; therefore, the two additional pancreatic foci were not yet known at the time of tissue acquisition and could not be targeted for biopsy. Following the PET/CT findings, the multidisciplinary tumor board concluded that the substantially lower SUV values of these two foci (25 and 10, compared with SUV max 95 of the primary lesion) were not consistent with independent neoplastic disease. A multidisciplinary tumor board reviewed the case and discussed surgical options, including duodenocephalopancreatectomy with pylorus-preserving (DCP-PP) versus total pancreatectomy. Moreover, a preoperative optimization through somatostatin analog therapy and supervised weight reduction was recommended.

### 2.4. Therapeutic Intervention

Bridging cytostatic therapy with long-acting octreotide (30 mg IM every 28 days) was administered for 10 cycles, in combination with a supervised weight-loss program. The treatment was well tolerated and resulted in a reduction in BMI from 34.6 to 30.7 kg/m^2^ over nine months. A restaging CT scan performed after approximately three months showed stable disease. Tumor markers remained negative (CEA < 2 ng/mL; CA 19–9 < 9 U/mL).

The patient subsequently underwent pylorus-preserving duodeno-pancreatico-jejunostomy with cholecystectomy two weeks later. Post-operative recovery was uneventful.

### 2.5. Histopathological Findings

Final histology revealed multifocal, well-differentiated NET G1 with intraductal extension into the dorsal duct, confirming pancreas divisum anatomy. The Ki-67 index was 1% and showed loss of DAXX expression, while ATRX expression was preserved, consistent with the preoperative EUS-guided fine-needle biopsy. Resection margins were negative. Focal vascular and perineural invasion were present, while no lymphatic invasion was identified.

## 3. Discussion

The growth of pNETs alongside the MPD is exceedingly rare, with fewer than 20 cases reported in the literature [1,5,6,7,8,9,10]. When present, this growth pattern can obstruct pancreatic outflow, leading to ductal dilatation and clinical or radiologic features that closely mimic IPMN or PDAC.

Walter et al. [8] and Kiyonaga et al. [10] described pNETs invading the MPD, raising the possibility that intraductal growth may represent a distinct biological subtype. In both cases the diagnosis was made after surgery. Compared with previously reported cases, our observation provides several additional insights. First, the intraductal growth pattern was recognized preoperatively rather than retrospectively on the surgical specimen. Second, the lesion arose in the setting of pancreas divisum and involved the dorsal pancreatic duct, an anatomical context that may further complicate radiologic interpretation and has rarely been described in association with pNETs. Finally, comprehensive preoperative characterization, including histologic grading and molecular assessment, was achieved through EUS-guided tissue acquisition. Beyond the rarity of intraductal extension in pNETs, this case underscores the central role of EUS in the preoperative diagnosis of atypical pancreatic lesions. EUS provides superior spatial resolution compared with cross-sectional imaging and is particularly effective in characterizing small or duct-related pancreatic tumors [11]. In intraductal lesions, EUS allows precise assessment of the relationship between the tumor and the pancreatic ductal system, often clarifying findings that remain ambiguous on CT or MRI. In our patient, EUS was essential to identify the intraductal growth within the dorsal duct and to confirm the underlying pancreas divisum anatomy, features that are difficult to appreciate radiologically. Equally important, EUS-FNB provided a tissue sample adequate for full histopathologic evaluation, with a grading that was fully concordant with the surgical specimen. Several studies have demonstrated the high diagnostic accuracy of EUS-FNB for pNET, including reliable Ki-67 assessment and strong concordance with final pathology [12,13,14].

Moreover, recent evidence indicates that EUS-FNB can also support molecular characterization. Mastrosimini et al. showed that EUS-FNB allows accurate evaluation of DAXX/ATRX expression and alternative lengthening of telomeres (ALT) status, with high agreement between cytologic samples and surgical specimens. These molecular alterations are increasingly recognized as markers of more aggressive tumor behavior and poorer prognosis in pancreatic neuroendocrine tumors [15]. In our patient, histological examination showed loss of DAXX expression, while ATRX expression was preserved.

This reinforces the value of EUS as a comprehensive preoperative tool, capable of informing risk stratification and guiding surgical decision-making, particularly in cases with atypical presentations such as intraductal growth.

The complexity of this case also highlights the importance of multidisciplinary management. The tumor board discussion allowed correlation of cross-sectional imaging, EUS morphology, cytologic and molecular findings, and the patient’s metabolic profile. Patients with metabolic syndrome may have an increased risk of pancreatic pathology; therefore, careful imaging evaluation may be warranted in selected individuals.

This collaborative approach was essential to coordinate preoperative medical and nutritional optimization, encompassing both somatostatin analog therapy and supervised weight-loss interventions, and to define the most appropriate surgical strategy, balancing oncologic radicality with preservation of pancreatic function. In particular, the decision to proceed with pylorus-preserving DCP rather than total pancreatectomy was supported by the absence of true multifocal disease and by the reliable preoperative characterization provided by EUS-FNB.

## 4. Conclusions

In summary, this case expands the spectrum of pNET presentations by documenting intraductal growth within the dorsal duct in the setting of pancreas divisum, an association not previously described. It illustrates how EUS and EUS-FNB are indispensable tools for the evaluation of atypical pancreatic lesions, providing high-resolution morphologic detail, accurate grading, and reliable molecular characterization. The integration of these findings within a multidisciplinary framework enabled precise diagnosis, preoperative optimization, and tailored surgical planning. Furthermore, this case highlights the importance of maintaining a high index of suspicion for pancreatic pathology in patients with metabolic syndrome, in whom a proactive imaging strategy may facilitate earlier diagnosis. This case also raises the possibility that intraductal extension may reflect a distinct biological behavior in a subset of pNETs, warranting further investigation.

## Figures and Tables

**Table 1:** Case reports of pNET with intraductal growth.

Title	Author, Year	Diagnostic Method	Final Diagnosis	Treatment
A Case of Pancreatic Neuroendocrine Tumor Growing Intraductal Extension toward the Main Pancreatic Duct Complicated by Thrombocytopenia: Diagnostic Challenges and Management Strategy	K. Soga et al. 2025 [5]	SPACE + Post-operative	NET G2 In MPD (body)	Pancreaticoduodenectomy
Pancreatic neuroendocrine tumor featuring growth into the main pancreatic duct and tumor thrombus within the splenic vein: a case report	T. Miyata et al. 2020 [6]	Post-operative	NET in MPD (body)	Total pancreatectomy
Pancreatic neuroendocrine neoplasm invading the entire main pancreatic duct diagnosed by a preoperative endoscopic biopsy	T. Kimura et al. 2020 [7]	Endoscopic biopsy	NET G3 in MPD (head)	Total pancreatectomy
Primary neuroendocrine tumors of the main pancreatic duct: a rare entity	T. Walter et al. 2011 [8]	Post-operative	5 cases: NET G1 in MPD	Surgical resection
Pancreatic neuroendocrine carcinoma with unique morphological features mimicking intraductal papillary mucinous carcinoma: A case report	H. Tanaka et al. 2022 [9]	POPS-biopsy	NEC in MPD (tail)	Supportive care
Pancreatic neuroendocrine tumor with extensive intraductal invasion of the main pancreatic duct: a case report	M. Kiyonaga et al. 2014 [10]	Post-operative	NET G2 in MPD (head)	Pancreaticoduodenectomy

SPACE: serial pancreatic juice aspiration cyrological examination; NET: neuroendocrine tumor MPD: main pancreatic duct; POPS: per-oral pancreatoscopy; NEC: neuroendocrine carcinoma.

## Data Availability

The original contributions presented in this study are included in the article. Further inquiries can be directed to the corresponding author.

## Abbreviations

The following abbreviations are used in this manuscript:

EUS: Endoscopic UltraSound. NET: neuroendocrine neoplasia. pNET: pancreatic neuroendocrine neoplasia. CT: computed tomography. PET: positron emission tomography. SUV: standardized uptake value. IPMN: intraductal papillary mucinous neoplasm. PDAC: pancreatic ductal adenocarcinoma. DCP P-P: duodenocephalopancreasectomy pylorus-preserving. SPACE: serial pancreatic juice aspiration cytological examination. POPS: per-oral pancreatoscopy. MEN1: multiple endocrine neoplasia type 1. VHL: von Hippel-Lindau. IM: intramuscular. GP: general practitioner.
